# Deep learning algorithm for detection of acute heart failure using standard ECG waveforms

**DOI:** 10.1093/ehjdh/ztaf132

**Published:** 2025-11-10

**Authors:** Sang Mee Lee, Taeyoung Kim, Mirae Shin, Jin-Oh Choi, Myung Jin Chung, Darae Kim

**Affiliations:** Department of Health Sciences and Technology, SAIHST, Sungkyunkwan University, Seoul, Republic of Korea; Medical AI Research Center, Research Institute for Future Medicine, Samsung Medical Center, Seoul, Republic of Korea; Medical AI Research Center, Research Institute for Future Medicine, Samsung Medical Center, Seoul, Republic of Korea; Division of Cardiology, Department of Medicine, Heart Vascular Stroke Institute, Samsung Medical Center, Sungkyunkwan University School of Medicine, Seoul, Republic of Korea; Division of Cardiology, Department of Medicine, Heart Vascular Stroke Institute, Samsung Medical Center, Sungkyunkwan University School of Medicine, Seoul, Republic of Korea; Department of Radiology and Medical AI Research Center, Samsung Medical Center, 81 Irwon-ro, Gangnam-gu, Seoul 06351, Republic of Korea; Department of Data Convergence and Future Medicine, Sungkyunkwan University School of Medicine, 81 Irwon-ro, Gangnam-gu, Seoul 06351, Republic of Korea; Division of Cardiology, Department of Medicine, Heart Vascular Stroke Institute, Samsung Medical Center, Sungkyunkwan University School of Medicine, Seoul, Republic of Korea

**Keywords:** Artificial intelligence, Electrocardiogram, Acute Heart failure

## Abstract

**Aims:**

To develop and evaluate a deep learning model for immediate and accurate diagnosis of acute heart failure(HF) using standard 12-lead electrocardiogram(ECG) waveforms collected from a large cohort of patients.

**Methods and results:**

We retrospectively analysed patients aged > 18 years who underwent transthoracic echocardiogram, *n*-terminal pro-B type natriuretic peptide (NT-proBNP) evaluation, and ECG within one week of clinical diagnosis at Samsung Medical from 1 February 2011 and 31 December 2022. The cohort included 1949 acute HF patients and a control group of 24 603 patients with normal NT-proBNP levels and no significant cardiac dysfunction. Four deep learning models (1D-CNN-Res, 1D-CNN-Dense, CRT-Net without transformer, CRT-Net) and their ensemble were developed using an 8:2 stratified split, ensuring no patient overlap. An external validation was performed using MIMIC-IV dataset, which comprised 7868 acute HF patients and 16 025 controls. The performance was evaluated using the area under the receiver operating characteristic curve (AUROC), sensitivity, specificity, positive predictive value (PPV), negative predictive value (NPV), and F1-score. The ensemble model demonstrated the best diagnostic performance with an AUROC of 0.997 and F1-score of 0.649 and the external validation showed AUROC of 0.842 and F1-score of 0.640. Notably, F1-score indicated diagnostic performance across a diverse range of ejection fraction values and demographic subgroups. Post-hoc analysis of false-positive cases revealed underlying cardiovascular risks, highlighting the model’s utility in identifying high-risk patients.

**Conclusion:**

The proposed deep learning models demonstrated remarkable performance in diagnosing acute HF. These findings support its potential utility in facilitating early diagnosis and improving clinical outcomes.

## Graphical Abstract

**Graphical Abstract:**
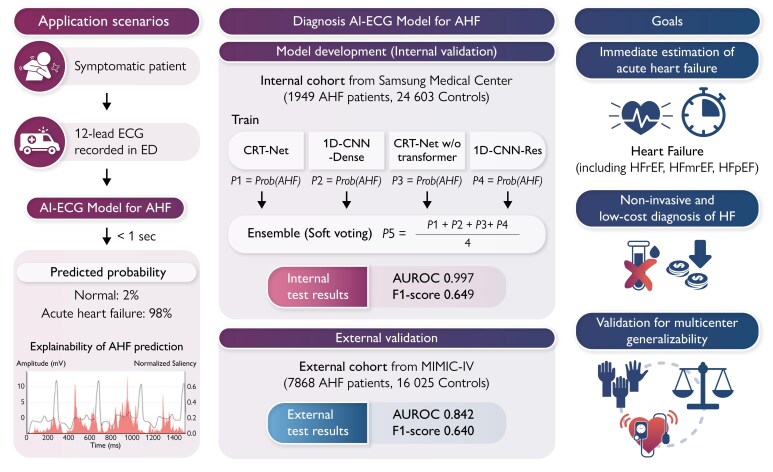

## Introduction

Acute heart failure (HF) is the leading cause of unexpected hospital admissions in elderly patients (age > 65 years), contributing to substantial increases in morbidity, mortality, and healthcare costs.^1–3^ Acute HF is prognostically important because of its association with high in-hospital and post-discharge mortality rates.^4^ Furthermore, survivors often face a substantially reduced quality of life and frequent acute hospital readmissions.

European Society of Cardiology (ESC) guidelines suggest suspicion of HF when a patient develops related symptoms, such as shortness of breath, fatigue, and leg swelling. However, these symptoms are non-specific and are shared with other comorbidities, such as chronic pulmonary lung disease, anaemia, or obesity. Physicians may misinterpret symptoms, and the diagnosis of acute HF can be delayed unless patients develop severe symptoms and are referred to specialists.^5^ Documenting structural and functional dysfunctions of the heart as the cause for the clinical presentation supports the diagnosis of HF through tests like echocardiography. However, half of HF patients have preserved left ventricular ejection fraction (EF). Because the diagnosis of HF requires comprehensive evaluation, including review of history, physical examination, laboratory tests, electrocardiogram, chest × ray, and cardiac imaging, delayed diagnosis or misdiagnosis can occur in the primary care setting.

In some cases, delay in diagnosis of acute HF can increases the risk of cardiogenic shock and multi-organ failure, underscoring the critical need for timely identification of acute HF in the clinical setting to initiate prompt intervention and improve patient outcomes.

Artificial intelligence–enabled electrocardiogram (AI-ECG) recently has proven to be a transformative force in healthcare, greatly enhancing the diagnostic process for cardiovascular disease.^6–8^ AI has significantly enhanced the application of ECG by detecting subtle abnormalities that might have been overlooked by traditional human analysis. This method uses machine learning algorithms that are trained on extensive datasets of cardiac signals and may facilitate the detection of acute HF from ECG with a level of precision that surpasses traditional techniques. As summarized in Supplementary material online, *Table S1*, many researchers have tried to diagnose HF with AI; however, most such studies have faced significant limitations.^9,10^ Previous studies mainly focused on specific HF categories, such as heart failure with reduced ejection fraction (HFrEF) or preserved ejection fraction (HFpEF), overlooking the continuous spectrum of ejection fraction values.^7,11^

In this study, we aimed to develop and evaluate the performance of AI-ECG across a comprehensive range of EF values, enabling inclusive and accurate assessments that capture the entire scope of HF. We also leveraged robust models that were previously validated in large-scale studies and enhanced their performance through an ensemble method to refine diagnostic accuracy and achieve superior results (*Figure*).^6,7,12^

## Patients and methods

### Study materials

We retrospectively analysed patients aged > 18 years who underwent transthoracic echocardiogram, *n*-terminal pro-B type natriuretic peptide (NT-proBNP) evaluation, and ECG within one week of clinical diagnosis at Samsung Medical from 1 February 2011 and 31 December 2022. Patients with incomplete ECG waveforms were excluded. Acute HF patients were identified as those who were presented with new or worsening symptoms of HF, including dyspnoea, fatigue, and fluid retention as a result of elevated cardiac filling pressures and systemic congestion. A total of 1949 acute HF patients was identified. Acute HF diagnosis was confirmed and treated by HF specialists. For the control group, we included individuals who underwent health screening at the health promotion centre of Samsung Medical Center with NTproBNP < 125 pg/mL,^5,13^ those without significant valvular heart disease (more than moderate regurgitation or mild stenosis), and those without LV systolic dysfunction (LVEF < 50%). A total of 24 603 patients with 31 834 ECG records was included as the control group. This study was approved by the Institutional Review Boards(IRB) of Samsung Medical Center.

### Data sources

For the internal dataset, raw aw 12-lead ECG waveforms, recorded digitally at 500 Hz using Philips ECG Machines and clinical information, were extracted from Samsung Medical Center’s Clinical Data Warehouse, DARWIN-C. The ECG data were stored as numerical time-series in CSV format. All samples were labelled by a board-certified cardiologist who did not participate in the data collection process.

For external validation, we used the publicly available MIMIC-IV database (v2.2), which includes de-identified health records from patients admitted to Beth Israel Deaconess Medical Center in Boston, Massachusetts. From this, we selected 7868 patients diagnosed with acute heart failure in the emergency department, identified using relevant diagnostic codes (see Supplementary material online, *Table S2*), and 16 025 control patients without a cardiovascular diagnosis during their admission.

### Data preprocessing

Each ECG signal was 10 s in length, comprising a total of 5000 data points. ECG signals were normalized using MinMaxscaler to a range of −1 to 1. In the internal dataset. We split the dataset 8:2 into training and test sets; records from 1 February 2011 to 31 December 2017 were used for the training set, and records from 1 January 2018 to 31 December 2022 were used for the test set. To avoid bias from using multiple ECGs from the same patient, we included only one ECG record per patient. For HF patients with multiple records in the study period, the most recent ECG was selected; for the control group, the first available ECG record was used. The overall data processing workflow is shown in *Figure 2*.

### Model development

**Figure 1:**
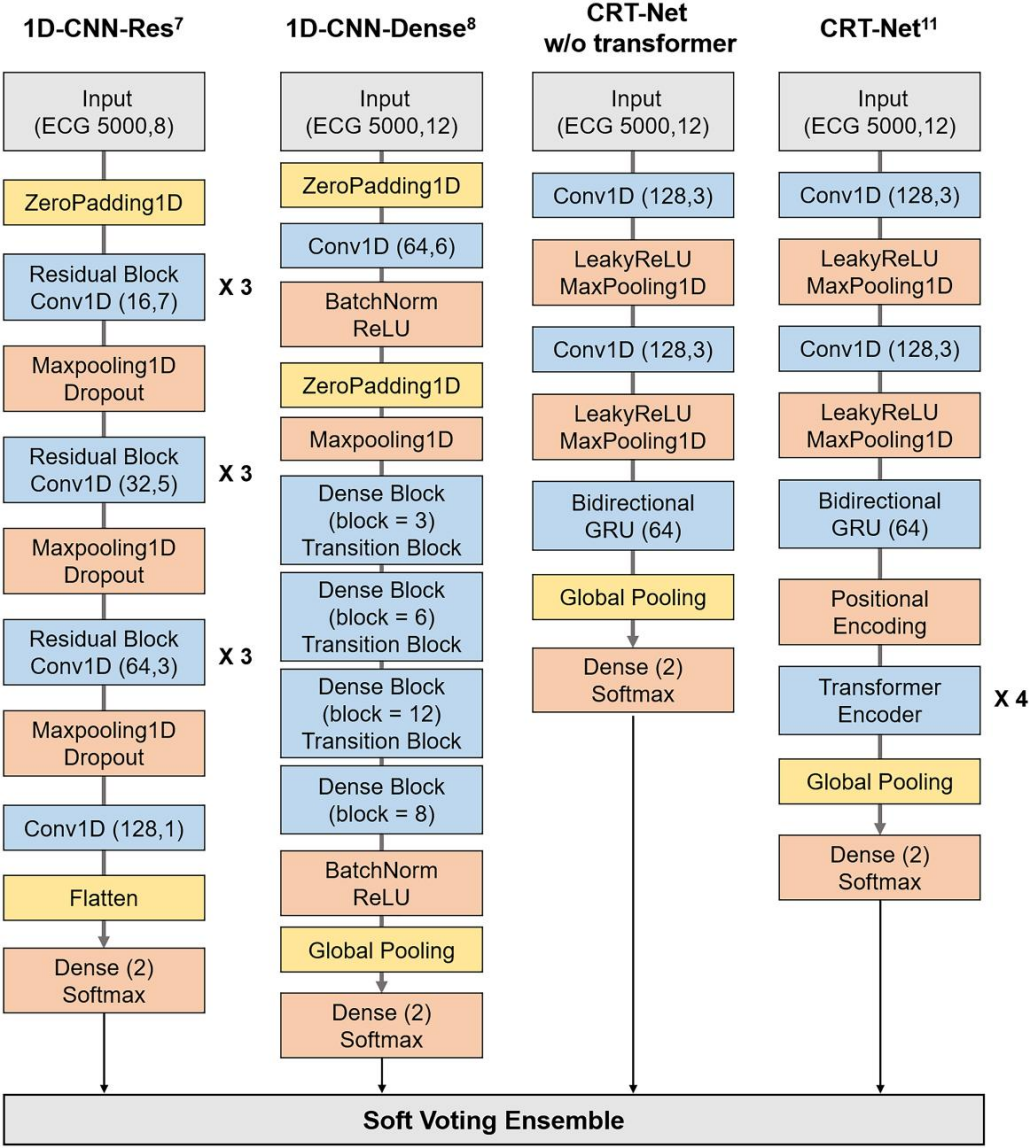
Deep learning model architecture for HF diagnosis the model architectures used in this study are shown: (*A*) 1D-CNN with residual blocks (1D-CNN-Res), (*B*) 1D-CNN with dense blocks (1D-CNN-Dense), (*C*) CRT-Net without transformer, and (*D*) CRT-Net.

For diagnosis of HF, we reproduced or modified existing neural architectures and compared the performances of five models: a 1D-convolutional neural network with residual blocks (1D-CNN-Res)^6^ (ZI Attia *et al*., 2019), a 1D-convolutional neural network with dense blocks (1D-CNN-Dense)^7^ (M Cohen-Shelly *et al*., 2021), CRT-Net^12^ (Liu *et al*., 2022) without a transformer, CRT-Net, and ensemble with soft average voting. A detailed comparison of these model architectures is shown in *Figure 1*. We directly analysed the raw waveform data from all ECGs without relying on quantified time-series features, such as QRS duration, QT, and RR interval. We used the Keras framework with a TensorFlow 2.7.0 and Python 3.7. Each model was trained with a maximum of 100 epochs, with a batch size of 16, a learning rate of 0.001 and early stopping was applied to prevent overfitting. Additionally, we determined the optimal threshold based on the Youden’s index in the internal evaluation. For external validation, a fixed threshold corresponding to 90% sensitivity was applied to better reflect real-world clinical practice.

1. 1D-CNN-Res^6^
  We adopted the 1D-CNN-Res classification model^6^ developed by Attia ZI *et al*. for our study. This model consists of six residual blocks, each containing 1D convolutional layers for feature extraction and shortcut connection to reduce the gradient vanishing problem.
2. 1D-CNN-Dense^7^
  We used the 1D-CNN-Dense classification model^7^ developed by Cohen-Shelly M *et al*. for our study. This model consists of four dense blocks with multiple layers of 1D convolutional operations. The dense connectivity patterns promote feature reuse and mitigate the vanishing gradient problem, while the design efficiently captures both local and global patterns in the ECG waveforms.
3. CRT-Net^12^ without transformer
  A model derived from CRT-Net^12^ by Lie *et al*. was developed, comprising convolutional neural networks (CNN) for local spatial feature extraction and gated recurrent units (GRU) for time-series feature capture, excluding the transformer encoders. We adopted this architecture after experimenting with various modifications to CRT-Net, where removal of the transformer module led to improved performance. The model also offers a simpler architecture compared with CRT-Net, which reduces both the complexity and computational cost.
4. CRT-Net^12^
  The CRT-Net,^12^ which combines convolutional neural network (CNN), bidirectional gated recurrent units (GRU), and transformer encoders, was used for recognition and classification of 1-D ECG signals. The early layers of CRT-Net consist of convolutional blocks and bi-directional GRU to capture patterns in both forward and reverse directions. Transformer encoders capture the long-range dependencies in ECG signals, and self-attention indicates the importance of different parts of the waveforms for extraction of features.

**Figure 2:**
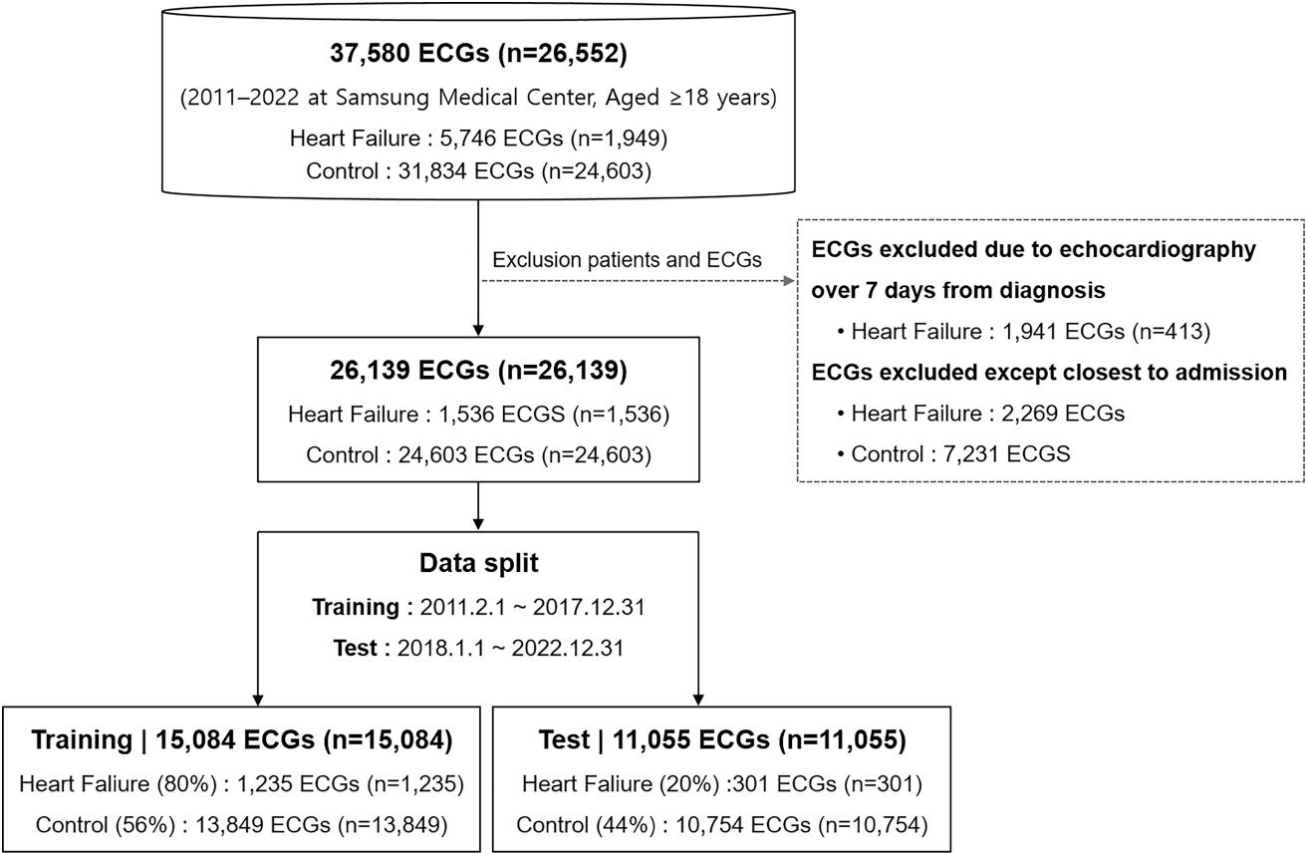
Flowchart of data processing.

### Performance evaluation

To evaluate the model performance, we used widely adopted metrics of area under the receiver operating characteristic curve (AUC), accuracy (Acc), sensitivity (Sen), specificity (Spe), positive predictive value (PPV), negative predictive value (NPV), and F1-score. These metrics were calculated based on the counts of true positives (TP), true negatives (TN), false positives (FP), false negatives (FN), and the total numbers of positive (P) and negative (N) samples.


$$\begin{array}{rl} \mathrm{AUC} & =\int_{0}^{1}\;\mathrm{TPR}(\mathrm{FPR})\;d\mathrm{FPR}\\ \mathrm{Acc} & =\frac{TP+TN}{TP+TN+FP+FN} \end{array}$$

$$\mathrm{Sen}(\mathrm{TPR})=\;\frac{TP}{TP+FN},\;\;\mathrm{Spe}=\;\frac{TN}{TN+FP}\;$$

$$\mathrm{FPR}=\;\frac{FP}{TN+FP}=1-Specificity$$

$$PPV=\frac{TP}{TP+FP},\;\;NPV=\;\frac{TN}{TN+FN}$$

$$F1\;Score=\;\frac{2TP}{2TP+FP+FN}$$

## Results

### Baseline characteristics of the acute HF cohort

In the overall group of HF patients, the mean length of time between the ECG and echocardiography was 7.5 h; more than 64.2% of the ECGs matched within 24 h of the index echocardiography. The clinical characteristics of the control and acute HF patients are summarized in *Table 1*. In the acute HF cohort, the median age was 78 years, and 58.6% of the patients were male. Approximately 20.5% had dilated cardiomyopathy and 55.8% had coronary artery disease. The median NT-proBNP and left ventricular EF were 4378 pg/mL and 30.5%, respectively. In the HF cohort, 59% were classified as HF with reduced EF, 9.5% were classified as HF with mildly reduced EF, and 31.5% were classified as HF with preserved EF.

**Table 1:** Baseline patient characteristics (N = 26 552)

	Acute HF (*n* = 1949)	Control (*n* = 24 603)	*P* value
**Demographics**			
Age, years	78 (65–86)	53 (48–59)	<0.05
Men, *n* (%)	1142 (58.6%)	16 579 (67.4%)	<0.05
BMI, kg/m^2^	23.2 (21.0–25.8)	24.4 (22.3–26.3)	<0.05
Systolic BP, mmHg	98 (88–113)	120 (110–133)	<0.05
**Co-morbidities (*n*, %)**			
Dilated cardiomyopathy	400 (20.5%)	153 (0.6%)	
Hypertension	1572 (80.7%)	10 505 (42.7%)	<0.05
Diabetes	1217 (62.4%)	7037 (28.6%)	<0.05
Coronary artery disease	1088 (55.8%)	331 (1.1%)	<0.05
Atrial fibrillation	551 (28.3%)	205 (0.8%)	<0.05
Valvular heart disease	890 (45.5%)	40 (0.2%)	<0.05
Congenital heart disease	28 (1.4%)	29 (0.1%)	<0.05
Chronic obstructive pulmonary disease	88 (4.5%)	124 (0.5%)	<0.05
Dyslipidaemia	690 (35.4%)	924 (3.8%)	<0.05
**Laboratories**			
Haemoglobin, g/dL	11.9 (10.2–13.6)	14.7 (13.7–15.6)	<0.05
HbA1c, %	6.5 (5.9–7.4)	5.6 (5.3–5.9)	<0.05
LDL-C, mg/dL	85 (64–109)	125 (102–150)	<0.05
BUN, g/dL	25.3 (15.3–30.3)	13.3 (10.9–15.2)	<0.05
Creatinine, g/dL	1.07 (0.8–1.4)	0.88 (0.75–1.0)	<0.05
eGFR, mL/1.73m^2^	64.7 (42.4–83.7)	87.2 (77.4–97.3)	<0.05
NT-proBNP, pg/mL	8785 (4364–10 713)	31.7 (13.2–43.2)	<0.05
LV EF (%)	30.5 (20.7–48)	65.4 (61–69)	<0.05
HFrEF	1149 (59%)	—	
HFmrEF	186 (9.5%)	—	
HFpEF	614 (31.5%)	—	
**Medications at admission**			
β-blockers	691 (35.5%)	249 (1.01%)	<0.05
Digoxin	345 (17.7%)	3 (0.0%)	<0.05
Antiarrhythmic agents	329 (16.9%)	30 (0.1%)	<0.05
Calcium channel blockers (non-DHP)	326 (16.7%)	147 (0.6%)	<0.05

BMI, body mass index; HbA1c: haemoglobin A1c; NT-proBNP, *n*-terminal pro natriuretic peptide; LV, left ventricle; EF, ejection fraction; EDD, end diastolic dimension;

### Diagnostic performance of AI models

**Figure 3:**
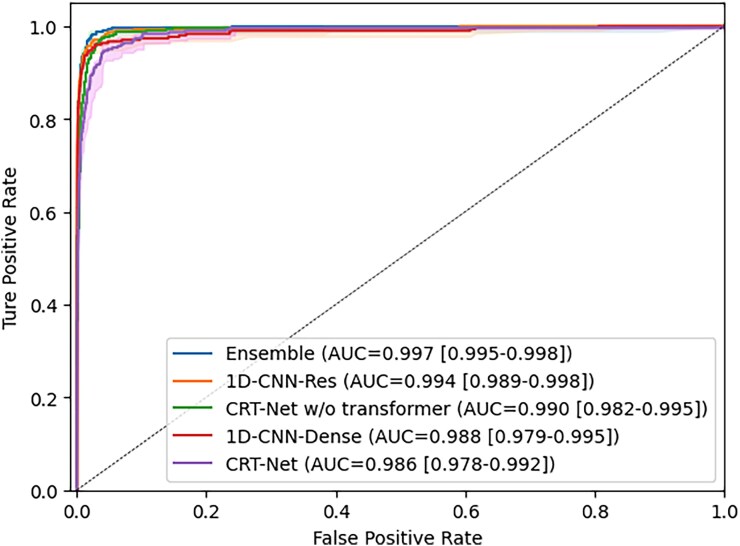
Receiver operating characteristic curves for deep learning models in HF diagnosis.

In the external validation cohort, various AI models using a total of 11 055 ECGs from the internal test set and 23 893 ECGs from the external test set (MIMIC-IV) with area under the receiver operating characteristic curve (AUROC), sensitivity, specificity, positive predictive value (PPV), negative predictive value (NPV), and F1-score (*Table 2*). The ensemble model demonstrated superior performance across most metrics. As shown in *Figure 3*, this model achieved the highest AUROC of 0.997 (95% confidence interval; 0.995–0.998) with sensitivity at 0.983 (95% CI; 0.968–0.997) and specificity at 0.975 (95% CI; 0.972–0.978). *Table 3* presents the confusion matrix for the ensemble model on the internal test set with an optimal balance between sensitivity and specificity.

**Table 2:** Performance of deep learning models for diagnosing HF

Test Data	Model	AUC	Sensitivity	Specificity	PPV	NPV	F1-Score
**Internal (*n*** **=** **11 055)**	CRT-Net	0.986 (0.978–0.992)	0.947 (0.922–0.971)	0.958 (0.955–0.962)	0.389 (0.353–0.426)	0.998 (0.998–0.999)	0.551 (0.514–0.585)
	1D-CNN-Dense	0.988 (0.979–0.995)	0.960 (0.936–0.981)	0.972 (0.969–0.975)	0.491 (0.450–0.529)	0.999 (0.998–1.000)	0.649 (0.613–0.685)
	CRT-Net without transformer	0.990 (0.982–0.995)	0.977 (0.958–0.991)	0.959 (0.955–0.963)	0.398 (0.361–0.433)	0.999 (0.999–1.000)	0.565 (0.528–0.601)
	1D-CNN-Res	0.994 (0.989–0.998)	0.970 (0.94–0.987)	0.974 (0.971–0.977)	0.512 (0.470–0.554)	0.999 (0.999–1.000)	0.670 (0.632–0.703)
	Ensemble	0.997 (0.995–0.998)	0.983 (0.968–0.997)	0.975 (0.972–0.978)	0.528 (0.485–0.570)	1.000 (0.999–1.000)	0.649 (0.610–0.687)
**External (*n*** **=** **23 893)**	CRT-Net	0.812(0.807–0.818)	0.900 (0.894–0.907)	0.464 (0.456–0.471)	0.452 (0.444–0.459)	0.904 (0.898–0.911)	0.602 (0.594–0.608)
	1D-CNN-Dense	0.815(0.809–0.820)	0.900 (0.894–0.907)	0.495 (0.487–0.503)	0.467 (0.459–0.475)	0.910 (0.903–0.916)	0.615 (0.607–0.623)
	CRT-Net without transformer	0.822(0.816–0.828)	0.900 (0.893–0.907)	0.478 (0.471–0.486)	0.459 (0.451–0.466)	0.907 (0.900–0.913)	0.608 (0.600–0.615)
	1D-CNN-Res	0.801(0.796–0.806)	0.900 (0.894–0.907)	0.554 (0.546–0.561)	0.498 (0.490–0.506)	0.919 (0.913–0.924)	0.641 (0.634–0.648)
	Ensemble	0.842(0.837–0.847)	0.900 (0.894–0.907)	0.551 (0.544–0.559)	0.496 (0.488–0.504)	0.918 (0.913–0.924)	0.640 (0.632–0.647)

The internal test set was derived from Samsung Medical Center, and the external test set was based on the MIMIC-IV database. All models were trained using only the internal training data. We determined thresholds for classification using Youden’s index for the internal test set and fixed at 90% sensitivity for the external test set. (95% CI; 95% Confidence Interval)

**Table 3:** Confusion matrix of the ensemble model with soft voting on the test set

	Predicted Value		
		AHF	Normal
Actual Value	AHF	296 (True Positives)	5 (False Negatives)
	Normal	265 (False Positives)	10 489 (True Negatives)

In external validation using the MIMIC-IV dataset, the ensemble model achieved the highest AUROC of 0.842 (95% CI: 0.837–0.847). When all models were evaluated at a fixed sensitivity of 0.90 to reflect clinical applicability, it showed the highest specificity of 0.551 (95% CI: 0.544–0.559).

### Post-hoc analysis of outcomes in the false positive group

To derive insights from the model’s decision on high-risk ECGs, we reviewed the long-term medical records of a subset of the false positive group to explore the potential cardiovascular risks. In the inference of the ensemble model on the test set, we identified 4 of 265 patients in the false positive group who were later found to have cardiovascular conditions. A detailed review of the 4 patients revealed that 3 were later diagnosed with coronary artery disease, while the other patient was diagnosed with atrial fibrillation (A-Fib) (see Supplementary material online, *Figure S1*). This finding highlights the model’s potential utility in identifying patients with underlying or developing cardiovascular conditions, despite their initial classification in the control group.

### Visual explanations

**Figure 4:**
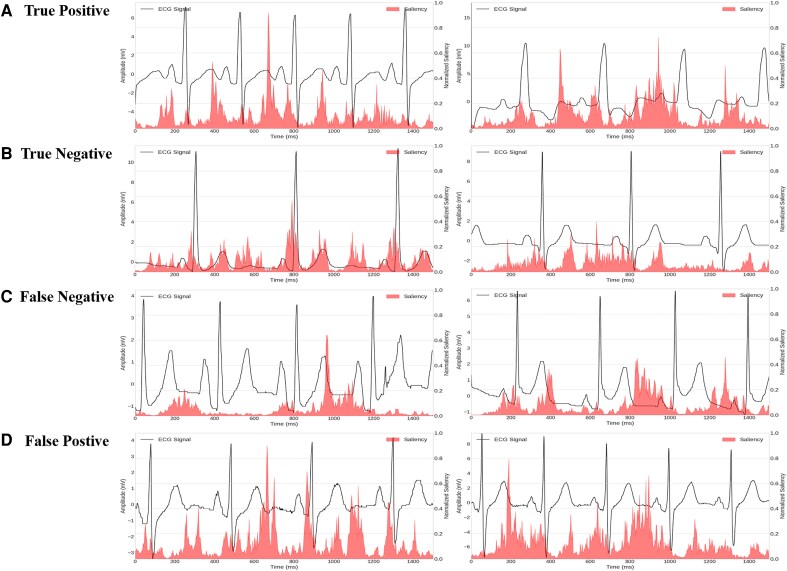
Visualization of ECG waveforms from 1D-CNN-res using a saliency map for HF prediction (*A*) true positive cases with clear activation in the T wave and TP segment (*B*) true negative cases with dispersed and low-saliency regions (*C*) false negative cases lacking consistent saliency, indicating reduced interpretability (*D*) false positive cases with focused activations despite no HF diagnosis, possibly reflecting subclinical abnormalities.

We used saliency mapping to visualize and highlight the regions in the ECG waveforms where the model identified abnormalities indicative of HF. *Figure 4* presents ECG waveforms from true positive (A), true negative (B), false negative (C), and false positive (D) cases as predicted by 1D-CNN-Res model, highlighting the model’s focus on different regions depending on the presence or absence of HF. *Figure 4(A)* shows true positive ECG waveforms where the model correctly predicted HF. The saliency map highlights regions with the highest saliency, particularly focusing on the diastole phase (T wave and TP segment). In contrast, *Figure 4(B)* shows true negative ECG waveforms where the model accurately predicted the absence of HF. These salience maps were more dispersed and showed lower saliency values than true positive cases. *Figure 4(C)* presents false negative cases in which the model failed to highlight consistent regions, indicating reduced interpretability in some borderline or ambiguous presentations of HF. *Figure 4(D)* illustrates false positive cases, where the model focused on distinct ECG segments despite the absence of HF, possibly reflecting subtle subclinical features or model overfitting. These visualizations demonstrate the model’s ability to differentiate between pathological and non-pathological ECGs and indicate the potential relevance of specific ECG features in the model’s decision-making process.

### Performance in different clinical subgroups

We examined the performance of the models across specific sex, age and subtypes.

### Sex and age subgroups

**Figure 5:**
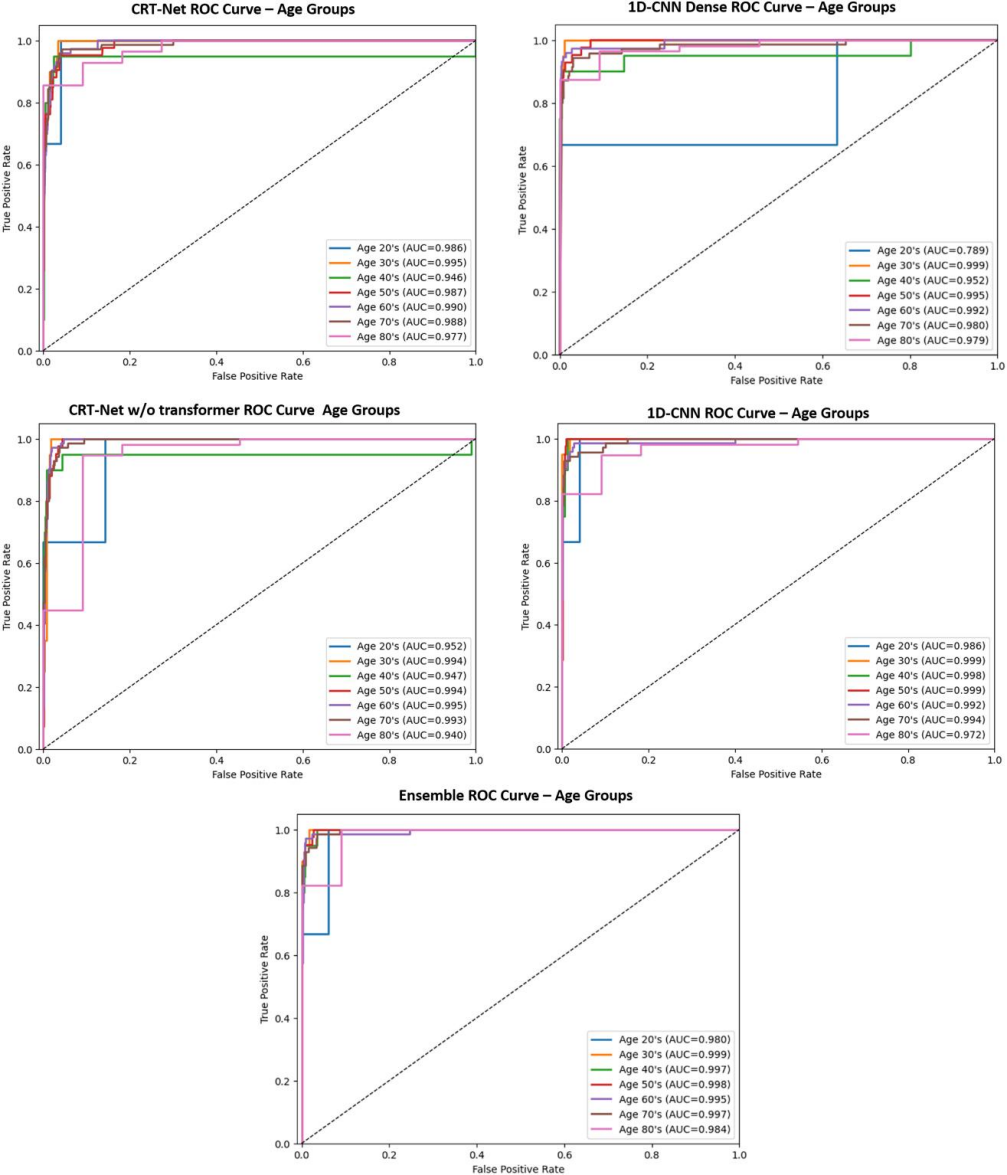
Receiver operating characteristic (ROC) curves for age subgroups: (*A*) CRT-Net, (*B*) 1D-CNN-Dense (*C*) CRT-Net w/o transformer, (*D*) 1D-CNN-Res, and (*E*) ensemble.

The distribution of control and heart failure cases by age in the internal test set is shown in Supplementary material online, *Table S3*. *Table 4* shows the performance of all models in terms of AUC, sensitivity, and specificity between male and female subgroups, with all scores over 0.93. Notably, the highest AUC for both females and males was observed in the ensemble model at 0.997. In contrast, the lowest AUC for females was 0.983 in CRT-Net, while that for males was 0.983 in 1D-CNN-Dense. Each model demonstrated robust performance across all groups, indicating their reliable diagnostic precision for HF [Supplementary material online, *Figure S2A*]. *Figure 5* presents the ROC curves for each model according to age groups on the test set. For the 60 s age group, which constituted the largest group among the HF patients, all models exhibited AUC scores above 0.99 (*Table 4*, *Figure 5*). The ensemble model achieved an AUC of 0.995, a sensitivity of 0.973, and a specificity of 0.992 (*Table 4*), demonstrating its reliable performance in this critical subgroup. This age group also showed the highest sensitivity across all models, demonstrating the ensemble model’s capability in effectively detecting HF not only in older patients, but across all age groups.

**Table 4:** Performance of each model by age group

Model	Age	10	20	30	40	50	60	70	80	90
**CRT-Net**	AUC	—	0.986	0.995	0.946	0.987	0.990	0.988	0.977	—
	Sensitivity	—	1.000	1.000	0.95	0.952	0.959	0.971	0.857	—
	Specificity	—	0.959	0.967	0.977	0.962	0.962	0.956	1.000	—
**1D-CNN-Dense**	AUC	—	0.789	0.999	0.952	0.995	0.992	0.980	0.975	—
	Sensitivity	—	0.667	1.000	0.900	1.000	0.973	0.943	0.862	—
	Specificity	—	1.000	0.990	0.993	0.930	0.972	0.969	1.000	—
**CRT-Net w/o transformer**	AUC	—	0.952	0.994	0.947	0.994	0.995	0.993	0.939	—
	Sensitivity	—	1.000	1.000	0.950	1.000	1.000	0.971	0.948	—
	Specificity	—	0.959	0.983	0.987	0.992	0.973	0.983	0.909	—
**1D-CNN-Res**	AUC	—	0.986	0.999	0.998	0.999	0.992	0.994	0.972	—
	Sensitivity	—	1.000	1.000	1.000	1.000	0.986	0.943	0.946	—
	Specificity	—	0.959	0.983	0.987	0.992	0.973	0.983	0.909	—
**Ensemble**	AUC	—	0.980	0.999	0.997	0.998	0.995	0.997	0.984	—
	Sensitivity	—	1.000	1.000	1.000	1.000	0.973	0.986	1.000	—
	Specificity	—	0.939	0.983	0.965	0.973	0.992	0.965	0.909	—

In the external validation cohort, a similar subgroup analysis was performed to evaluate the model’s generalizability (*Table 5*). Among younger patients (<50 years), males demonstrated better performance, with an AUROC of 0.857 compared to females. In the 50–64 age group, both sexes showed high AUROC values exceeding 0.84, with males again exhibiting higher specificity and precision. For patients aged ≥65 years, the model achieved an F1-score of 0.800 in males and 0.747 in females.

**Table 5:** Performance of the ensemble AI-ECG model in subgroups stratified by age and sex in the external validation cohort

Group	*n* (23 893) (HF/Control)	AUC	Sensitivity	Specificity	PPV	NPV	F1-score
Female (age <50)	4267 (126/4141)	0.839 (0.798–0.876)	0.706 (0.628–0.788)	0.839 (0.827–0.850)	0.118 (0.096–0.141)	0.989 (0.986–0.993)	0.202 (0.168–0.238)
Male (age <50)	4085 (229/3856)	0.882 (0.857–0.904)	0.838 (0.788–0.880)	0.767 (0.753–0.780)	0.176 (0.154–0.201)	0.988 (0.984–0.992)	0.291 (0.260–0.323)
Female (age 50–64)	3006 (507/2499)	0.835 (0.815–0.854)	0.799 (0.764–0.832)	0.725 (0.706–0.742)	0.371 (0.341–0.400)	0.947 (0.936–0.955)	0.506 (0.476–0.537)
Female (age 50–64)	2957 (754/2203)	0.842 (0.825–0.859)	0.706 (0.673–0.739)	0.844 (0.829–0.859)	0.607 (0.575–0.640)	0.893 (0.880–0.906)	0.653 (0.625–0.679)
Female (age >65)	5322 (3313/2009)	0.774 (0.761–0.786)	0.696 (0.681–0.711)	0.721 (0.702–0.741)	0.805 (0.790–0.818)	0.590 (0.571–0.609)	0.747 (0.734–0.758)
Male (age >65)	4256 (2939/1317)	0.819 (0.806–0.832)	0.738 (0.721–0.753)	0.761 (0.736–0.786)	0.873 (0.860–0.887)	0.565 (0.541–0.588)	0.800 (0.789–0.811)

95% confidence intervals are shown in parentheses. The optimal thresholds were selected according to the maximum Youden’s index

We evaluated model performance in internal test dataset across heart failure subtypes defined by left ventricular ejection fraction (LVEF). As summarized in *Table 6*, the ensemble model maintained high sensitivity for all subtypes: 0.986 for HFrEF (LVEF < 40%), 0.944 for HFpEF (LVEF ≥ 50%), and 0.885 for HFmrEF (LVEF 40–49%). Notably, the false negative rate was highest in the HFmrEF group (11.5%), suggesting a potential challenge in identifying this borderline population. These findings emphasize the importance of continued refinement to enhance diagnostic accuracy in subtypes with less distinctive ECG features.

**Table 6:** Sensitivity and false negative rate of the ensemble model by heart failure subtype

Subtype	LVEF range	*n*	TP	FN	Sensitivity	FN rate (%)
HFrEF	LVEF < 40%	219	216	3	0.986	1.37
HFmrEF	LVEF 40–49%	26	23	3	0.885	11.50
HFpEF	LVEF ≥ 50%	54	51	3	0.944	5.56

HFrEF, heart failure with reduced ejection fraction; HFmrEF, heart failure with mildly reduced ejection fraction; HFpEF, heart failure with preserved ejection fraction; LVEF, left ventricular ejection fraction; TP, true positive; FN, false negative.

## Discussion

In this study, we developed and validated AI-ECG models to diagnose HF patients regardless of left ventricular EF. The AI-ECG algorithm demonstrated satisfactory performance for diagnosing HF patients regardless of sex and age.

HF is a common diagnosis in primary health care. It is one of the main causes of morbidity and mortality worldwide. HF is usually suspected as a result of characteristic signs and symptoms such as dyspnoea, fatigue, and peripheral oedema, which are non-specific and can be caused by other conditions, complicating clinical diagnosis. A cross-sectional study found that more than one-third (36.5%) of patients with a diagnosis of HF from primary care physicians did not actually have definite HF as defined by the European Society of Cardiology (ESC) guidelines, indicating significant overdiagnosis.^14^ Underdiagnosis is also a concern, as studies have shown that many patients with suspected HF do not receive recommended diagnostic tests like electrocardiograms or echocardiograms in primary care.^15,16^ Adherence to evidence-based guidelines for diagnosing HF in primary care is suboptimal, with only 7% of patients following the recommended pathway. Factors such as older age, presence of comorbidities, and socioeconomic deprivation are associated with lower adherence to guidelines and potentially missed or delayed HF diagnosis. Therefore, our AI-ECG algorithm will be helpful and is expected to increase the rate of correct diagnosis of HF.

In particular, this model is designed to use routinely available clinical variables at the time of admission, enabling early risk stratification without the need for additional testing. In a real-world setting, the model output can be integrated into the electronic medical record (EMR) system to provide automated alerts or flag high-risk patients for closer monitoring. Such decision support can assist clinicians in early triage, timely intervention planning, and shared decision-making, particularly in resource-limited settings or high patient-volume environments. To optimize model performance, we utilized a soft-voting ensemble of multiple architectures, including 1D-CNN-Res, 1D-CNN-Dense, CRT-Net, and CRT-Net without transformers. We adopted the ensemble to enhance prediction robustness by mitigating architecture-specific limitations and reducing the likelihood of misclassification. This approach proved especially effective in external validation, highlighting its suitability for real-world deployment in heterogeneous clinical populations.

In the external validation cohort, patients aged ≥65 years showed lower NPVs compared with younger subgroups. This finding is largely attributable to the higher prevalence of HF in this age group, which reduces NPV even in well-performing models. To aid interpretation, subgroup sample sizes were reported (*Table 5*). Importantly, although NPV was reduced, the model maintained diagnostic utility in older patients, with preserved PPV and F1-scores. These results indicate that threshold selection may need to be tailored to patient age, sex, and clinical context, reflecting the inherent trade-offs between sensitivity, specificity, PPV, and NPV in real-world practice.

Furthermore, the use of 1D CNN models for HF classification provided valuable insights into the model’s decision-making process. By applying activation mapping, we identified specific regions of ECG waveforms prioritized by the model. This interpretability aligns with clinical expertise and helps bridge the gap between machine learning outputs and medical decision-making.

Our visual mapping analysis, shown in *Figure 4*, provides intriguing insights into the ECG segments crucial for HF diagnosis. The saliency map generated by our 1D-CNN-Res model demonstrates the varying importance of different ECG components in diagnosing HF. Specifically, the saliency map highlights the significance of the T waves and TP segments. The T wave represents the repolarization of the ventricles. Abnormalities in T wave morphology may indicate an abnormality in myocardial repolarization.

In HF, T wave abnormalities such as flattening, inversion, or biphasic morphology are frequently observed. These changes may arise from repolarization heterogeneity due to increased wall stress, ischaemia, or neurohormonal activation, and are more prevalent in left ventricular hypertrophy or reduced EF. Our model may have detected these subtle, spatially-distributed repolarization patterns, particularly in the lateral leads (e.g. V5–V6, I, aVL), which are often underemphasized in routine interpretation but relevant to ventricular function. The model may have detected subtle variations in T wave characteristics that are indicative of underlying ventricular dysfunction. HF is accompanied by a range of electrophysiological changes, including delayed afterdepolarizations and prolonged action potentials. These changes can manifest in the ECG as alterations in the T wave and TP segment. The TP segment is the isoelectric period between the end of the T wave and the beginning of the *P* wave and reflects the balance between sympathetic and parasympathetic tone. Although often overlooked, the TP segment reflects autonomic tone and atrial electrical recovery. In HF, increased sympathetic activity and reduced vagal tone can lead to a shorter TP interval and dynamic baseline shifts. These subtle variabilities may not cross clinical thresholds of abnormality, yet may be detected by deep learning models through cumulative waveform deformations. Therefore, TP segment alterations could serve as indirect markers of neurohormonal imbalance in acute decompensated HF. While our saliency analysis highlighted the diastolic phase, it is plausible that other ECG features such as QRS widening, prolonged QTc, or PR interval variations may also contribute to the model’s decision-making. These parameters often reflect conduction abnormalities or structural remodelling, both of which are prevalent in advanced HF. Further saliency analyses focusing on these intervals could refine interpretability and guide clinical validation. These findings suggest that the AI-ECG model may offer diagnostic value beyond binary classification, by identifying latent ECG markers of haemodynamic stress and autonomic imbalance. From a clinical perspective, this enhances its potential role not only in diagnosis but also in risk stratification or early screening, particularly in primary care or pre-hospital settings.

Clinical interpretation of the four representative cases in *Figure 4* revealed that TP and TN patients generally exhibited consistent clinical risk profiles aligned with the model’s prediction. FP cases often involved patients with multiple comorbidities (e.g. older age, CKD) that warranted cautious prediction, whereas FN cases frequently lacked overt clinical indicators of risk or involved atypical presentations. These findings underscore the importance of complementing model output with clinician judgment, especially for borderline cases

The proposed AI-ECG model, initially developed for acute heart failure diagnosis, may also serve as a foundation for future applications aimed at predicting acute hospital admissions due to decompensated heart failure or differentiating between stable and unstable HF phenotypes. Such extensions could further enhance the clinical utility of the model across both acute care and longitudinal management settings.

The model’s emphasis on these specific ECG regions may reflect subtle changes in cardiac electrical activity, possibly related to ventricular repolarization and diastolic function, which are crucial in HF pathophysiology but often overlooked in traditional ECG analysis. This visualization not only supports the predictive capabilities of our model, but also opens up new avenues for research into the relationship between specific ECG patterns and the complex mechanisms underlying HF, particularly in its early stages or in cases of diastolic dysfunction.

## Limitations

This study has several limitations. First, it was conducted at a single tertiary referral medical centre, which may introduce referral bias. Second, our cohort comprised patients hospitalized with acute HF; we did not prespecify a comparison group of patients with stable chronic HF without recent worsening, nor did we analyse serial/baseline ECGs. Accordingly, the specificity for distinguishing acute from stable chronic HF could not be assessed, and future validation in clinically stable chronic HF populations and in serial-ECG (delta-ECG) designs is warranted. Third, medication ascertainment was incomplete for a subset of patients. Medication lists were abstracted at emergency department arrival and from in hospital orders; outpatient prescriptions from other institutions were variably available and, in some cases, unavailable. In addition, some drugs may have been temporarily withheld around the time of decompensation. Consequently, chronic medications exposure may be partially misclassified, which limit the interpretation of any associations between baseline medications use and electrocardiographic findings.

## Conclusion

The AI-ECG algorithm demonstrated acceptable performance in diagnosing HF in a real-world cohort. An ensemble model combining top-performing deep learning architectures from prior studies enhanced diagnostic accuracy, particularly in identifying HF across a wide range of EF values. This study underscores the potential of AI-ECG as a valuable tool in the early and accurate diagnosis of HF, which is critical in improving patient outcome and ultimately improving cardiovascular care.

## Supplementary Material

ztaf132_Supplementary_Data

## Data Availability

The data cannot be publicly shared due to restrictions imposed by our institutional review board approval. Deidentified test set data may be made available to researchers under a data use agreement after the study is published in a peer-reviewed journal.
