# Supplementary material for: Deep learning algorithm for detection of acute heart failure using standard ECG waveforms
Source: Eur Heart J Digit Health. 2025 Nov 10;7(2):ztaf132. doi: 10.1093/ehjdh/ztaf132 (PMC12853116; doi:10.1093/ehjdh/ztaf132)
Supplement: ztaf132_Supplementary_Data [file ztaf132_supplementary_data.zip › Supplementary Tables.docx]

**Supplementary Table 1**. Summary of previous AI-ECG studies for heart failure diagnosis

| Author, Year | Objective | Limitations |
| --- | --- | --- |
| Cohen-Shelly et al., 2021 | Develop an AI-based ECG screening tool to detect aortic valve stenosis | Not directly focused on heart failure; limited generalizability to broader HF populations and reliance on ECG features |
| Yasmin et al., 2021 | Review of AI applications in heart failure diagnosis | Broad review rather than a specific diagnostic study; lacks focus on a single HF category; discusses potential but not performance |
| Choi et al., 2022^10^ | Develop a deep learning algorithm (DeepECG-HFrEF) using ECG waveforms to identify HFrEF | Focused on HFrEF only; no external validation; limited applicability to HFpEF or acute HF; |
| Kwon et al., 2021 | Early detection of HFpEF using AI model based on ECG features | Targeted only HFpEF; small dataset; no external validation; limited generalizability; |

**Supplementary Table 2. ICD Codes for Identifying Acute Heart Failure Patients in the MIMIC-IV Dataset for External Validation**

| ICD Code | ICD Version | Diagnosis Description |
| --- | --- | --- |
| 42821 | ICD-9 | Acute systolic heart failure |
| 42823 | ICD-9 | Acute on chronic systolic heart failure |
| 42831 | ICD-9 | Acute diastolic heart failure |
| 42833 | ICD-9 | Acute on chronic diastolic heart failure |
| 42841 | ICD-9 | Acute combined systolic and diastolic HF |
| 42843 | ICD-9 | Acute on chronic combined HF |
| I5021 | ICD-10 | Acute systolic heart failure |
| I5023 | ICD-10 | Acute on chronic systolic heart failure |
| I5031 | ICD-10 | Acute diastolic heart failure |
| I5033 | ICD-10 | Acute on chronic diastolic heart failure |
| I5041 | ICD-10 | Acute combined systolic & diastolic HF |
| I5043 | ICD-10 | Acute on chronic combined HF |
| I50811 | ICD-10 | Acute right heart failure |
| I50813 | ICD-10 | Acute on chronic right heart failure |
| I501 | ICD-10 | Left ventricular failure, unspecified |
| 42830 | ICD-9 | Diastolic HF, unspecified |
| 42831 | ICD-9 | Acute diastolic heart failure |
| 42832 | ICD-9 | Chronic diastolic heart failure |
| 42833 | ICD-9 | Acute on chronic diastolic heart failure |
| I503 | ICD-10 | Diastolic heart failure |
| I5030 | ICD-10 | Diastolic HF, unspecified |
| I5031 | ICD-10 | Acute diastolic heart failure |
| I5032 | ICD-10 | Chronic diastolic heart failure |
| I5033 | ICD-10 | Acute on chronic diastolic heart failure |
| 42840 | ICD-9 | Combined HF, unspecified |
| 42841 | ICD-9 | Acute combined systolic and diastolic HF |
| 42842 | ICD-9 | Chronic combined HF |
| 42843 | ICD-9 | Acute on chronic combined HF |
| I504 | ICD-10 | Combined systolic and diastolic HF |
| I5040 | ICD-10 | Combined HF, unspecified |
| I5041 | ICD-10 | Acute combined systolic and diastolic HF |
| I5042 | ICD-10 | Chronic combined HF |
| I5043 | ICD-10 | Acute on chronic combined HF |

**Supplementary Table 3.** Distribution of Control and Heart Failure Cases by Age in the Test Set

| **Age** | **Control** | **Heart Failure** | **Total** |
| --- | --- | --- | --- |
| 10 | 3 | 0 | 3 |
| 20 | 49 | 3 | 52 |
| 30 | 300 | 20 | 320 |
| 40 | 2,761 | 20 | 2,781 |
| 50 | 4,491 | 42 | 4,533 |
| 60 | 2,680 | 73 | 2,753 |
| 70 | 459 | 70 | 529 |
| 80 | 11 | 58 | 69 |
| 90 | 0 | 15 | 15 |
|  | 10,754 | 301 | 11,055 |
