# Supplementary figures and images for: Deep learning algorithm for detection of acute heart failure using standard ECG waveforms
Source: Eur Heart J Digit Health. 2025 Nov 10;7(2):ztaf132. doi: 10.1093/ehjdh/ztaf132 (PMC12853116; doi:10.1093/ehjdh/ztaf132)

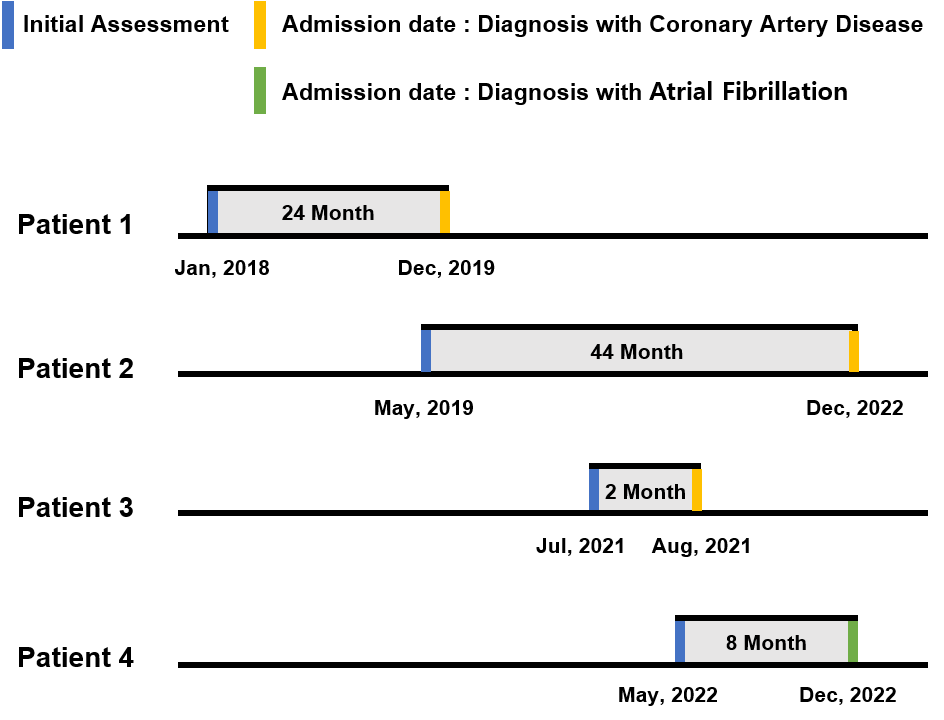

Supplement: ztaf132_Supplementary_Data [file ztaf132_supplementary_data.zip › Sup Fig 1.tif]

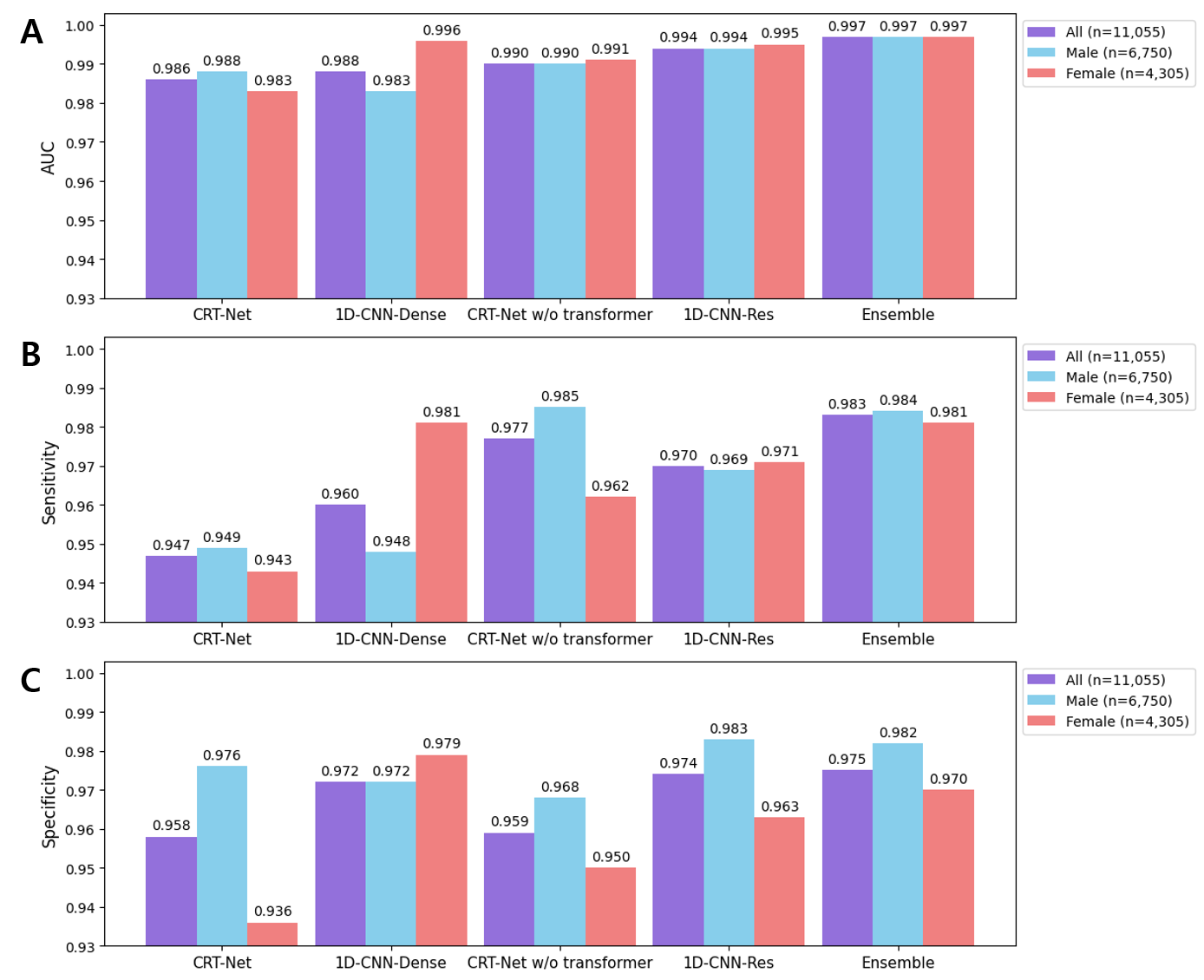

Supplement: ztaf132_Supplementary_Data [file ztaf132_supplementary_data.zip › Sup Fig 2.tif]
